# Supplementary material for: LncRNA NEAT1 protects uremic toxin-induced intestinal epithelial barrier injury by regulating miR-122-5p/Occludin axis
Source: PLoS One. 2025 May 8;20(5):e0322989. doi: 10.1371/journal.pone.0322989 (PMC12061087; doi:10.1371/journal.pone.0322989)
Supplement: Attachment — Submitted filename: Response letter.docx [file pone.0322989.s003.docx]

Dear reviewer,

Thank you very much for your comments and professional -advice. These opinions help to improve academic rigor of our article. Based on your suggestion and-request, we have made corrected modifications on the revised manuscript. Furthermore, we would like to show the details as follows

Question1

When submitting your revision, we need you to address these additional requirements.

Please ensure that your manuscript meets PLOS ONE's style requirements, including those for file naming. The PLOS ONE style templates can be found at https://journals.plos.org/plosone/s/file?

id=wjVg/PLOSOne_formatting_sample_main_body.pdfandhttps://journals.plos.org/plosone/s/file?id=ba62/PLOSOne_formatting_sample_title_authors_affiliations.pdf.

The author’s answer:

We have revised our manuscript as requested by PLOS One.

Question2

We note that you have indicated that there are restrictions to data sharing for this study. For studies involving human research participant data or other sensitive data, we encourage authors to share de-identified or anonymized data. However, when data cannot be publicly shared for ethical reasons,we allow authors to make their data sets available upon request. For information on unacceptable data access restrictions, please see<http://journals.plos.org/plosone/s/data-availability#loc-unacceptable-data-access-restrictions.>

The author’s answer:

We have chosen to upload the raw data to PLOS One as supporting information.

Question3

Please include your full ethics statement in the ‘Methods’ section of your manuscript file. In your statement, please include the full name of the IRB or ethics committee who approved or waived your study, as well as whether or not you obtained informed written or verbal consent. If consent was waived for your study, please include this information in your statement as well.

The author’s answer:

We have included the full ethics statement in the Methods section of the manuscript.

Question4

Please include a separate caption for each figure in your manuscript.

The author’s answer:

We have included a separate caption for each figure in our manuscript.

Question5

PLOS ONE now requires that authors provide the original uncropped and unadjusted images underlying all blot or gel results reported in a submission’s figures or Supporting Information files. This policy and the journal’s other requirements for blot/gel reporting and figure preparation arehttps://journals.plos.org/plosone/s/figures#loc-preparing-figures-from-image-files. When you submit your revised manuscript, please ensure that your figures adhere fully to these guidelines and provide the original underlying images for all blot or gel data reported in your submission. See the following link for instructions on providing the original image data:https://journals.plos.org/plosone/s/figures#loc-original-images-for-blots-and-gels.

The author’s answer:

We provide raw uncropped and unadjusted images of all blot or gel results in the Supporting Information file.

Additional questions

The figure quality is not appropriate. Reviewers have raised critical concerns regarding experiment set up and conclusions drawn. Authors need to adress these concerns and add a limitations section to the conclusions.In the current form this study is not of publication standard.

The author’s answer:

We have modified the quality of the figure.

Reviewer #1: This study aims to investigate the mechanism of lncRNA Nuclear Enriched Abundant Transcript 1 (NEAT1) in uremic toxin-induced intestinal epithelial barrier injury using Caco-2 and HEK293 cells. The findings demonstrate that lncRNA NEAT1 protects against uremic toxin-induced intestinal epithelial barrier injury by regulating the miR-122-5p/Occludin axis. However, this study has several limitations. It does not provide practical guidance on selecting urea treatment at 144 mg/dL for 48 hours, nor does it identify an optimal model for testing CKD recipient matches. Additionally, it lacks an in-depth analysis of the mechanisms underlying uremic toxin-induced intestinal epithelial injury.
1. The immunofluorescence images presented in this manuscript lack the clarity and resolution necessary for occludin staining (Figures 2G, 2H, 2I, 4F, and 5C).
2. In Figure 1, Western blot images D and H appear to be identical, particularly for ZO-1, claudin-1, and GAPDH. Could you clarify why these images seem to be duplicated?
3. Finally, the conclusion should address the debatable reduction of the tight junction proteins Occludin, ZO-1, and Claudin-1. Additionally, the permeability of sodium fluorescein does not appear to be significantly increased in the model group, which raises questions about the validity of the proposed mechanism involving the miR-122-5p/Occludin axis in supporting the observed injury.

The author’s answer:

Our study does have certain limitations. We reviewed the literature on the concentration and duration of urea intervention in intestinal epithelial cells, but there are no exact guidelines, and there may be different cell lines and types of uremic toxins used in different laboratories.In addition, the lack of in-depth analysis of the mechanism of uremic toxin-induced intestinal epithelial injury in this study is a major drawback. This is also where the research group needs to continue to explore.

1. We have modified the immunofluorescence images for the required clarity and resolution (Figure 2G, 2H, 2I, 4F, and 5C).
2. In Figure 1, images of western blots for ZO-1, claudin-1, and GAPDH are not duplicated, and we have provided untrimmed images in the Supplementary material.
3. The permeability of fluorescein sodium was significantly increased in the model group, and we have provided raw data along with statistical analysis in the Supplementary material.

Reviewer #2:

This manuscript investigates the role of LncRNA NEAT1 in urea induced Intestinal Epithelial barrier. Authors further explore the interaction between NEAT1 and miRNA-122-5p.This manuscript requires major revision before it could be accepted for publication. The language is not very easy to comprehend and would require significant change.

Authors have provided complete data that they have discussed in the paper. However, their final conclusion that "NEAT1 acts as a sponge of miRNA-1225p to regulate Occludin expression" need more investigation. Figure5B shows that overexpression of LV-NEAT1 in the presence of miR-122-5p mimic and urea fails to rescue expression of Occludin, which doesnt support their conclusion.I recommend that authors should evaluate expression of Occludin in a dose-dependent response for both miR-122-5p mimic and urea independently, In the presence of a fixed concentration of other molecule and overexpression of LV-NEAT1.

The author’s answer:

Dear reviewer, Figure 5B shows that LV-NEAT1 significantly upregulated Occludin expression and miR-122-5p mimic significantly downregulated Occludin expression. The expression of Occludin in the LV-NEAT1+miR-122-5p group was significantly higher than that in the miR-122-5p group, and overexpression of NEAT1 could rescue the down-regulation of Occludin caused by miR-22-5p mimics group. We have identified this significant difference in Figure 5B.

We appreciate for Reviewers'warm work earnestly, and hope the correction will meet with approval. Once again, thank you very much for your comments and suggestions.
