# Supplementary material for: LncRNA NEAT1 protects uremic toxin-induced intestinal epithelial barrier injury by regulating miR-122-5p/Occludin axis
Source: PLoS One. 2025 May 8;20(5):e0322989. doi: 10.1371/journal.pone.0322989 (PMC12061087; doi:10.1371/journal.pone.0322989)
Supplement: Attachment — Submitted filename: Response to Reviewers.docx [file pone.0322989.s004.docx]

Dear reviewer,

Thank you very much for your comments and professional -advice. These opinions help to improve academic rigor of our article. Based on your suggestion and-request, we have made corrected modifications on the revised manuscript. Furthermore, we would like to show the details as follows：

As some references were retracted, we have replaced them to ensure the accuracy of the article, which has been standardized in the article.

Reviewer #1: I still have some concerns regarding the manuscript. For the Western blots, please ensure the molecular weight (kDa) is specified for each protein. Additionally, clarify how the Western blot bands were quantified, and ensure this is clearly described. Specify the number of experiments conducted. Lastly, there are additional references missing in the manuscript that should be included in the final version.

My answer:Thanks for your suggestion. We have annotated the molecular weight of each protein in the figures of the article.The gray value of the target protein bands was quantitatively analyzed by imageJ, and each experiment was repeated 3 times to determine the expression level of the target protein.AdditionIy, we have refined and revised the references for completeness and accuracy.

Reviewer #3: The author has carefully considered the comments and revised the manuscript to enhance its scientific validity and transparency.

My answer:Thank you for your review. We tried our best to improve the manuscript and made some changes to the manuscript. These changes will not influence the content and framework of the paper.
